# Power laws in citation distributions: evidence from Scopus

**DOI:** 10.1007/s11192-014-1524-z

**Published:** 2015-01-22

**Authors:** Michal Brzezinski

**Affiliations:** Faculty of Economic Sciences, University of Warsaw, Dluga 44/50, 00-241 Warsaw, Poland

**Keywords:** Citation distribution, Power law, Statistical modelling, Scopus

## Abstract

Modeling distributions of citations to scientific papers is crucial for understanding how science develops. However, there is a considerable empirical controversy on which statistical model fits the citation distributions best. This paper is concerned with rigorous empirical detection of power-law behaviour in the distribution of citations received by the most highly cited scientific papers. We have used a large, novel data set on citations to scientific papers published between 1998 and 2002 drawn from Scopus. The power-law model is compared with a number of alternative models using a likelihood ratio test. We have found that the power-law hypothesis is rejected for around half of the Scopus fields of science. For these fields of science, the Yule, power-law with exponential cut-off and log-normal distributions seem to fit the data better than the pure power-law model. On the other hand, when the power-law hypothesis is not rejected, it is usually empirically indistinguishable from most of the alternative models. The pure power-law model seems to be the best model only for the most highly cited papers in “Physics and Astronomy”. Overall, our results seem to support theories implying that the most highly cited scientific papers follow the Yule, power-law with exponential cut-off or log-normal distribution. Our findings suggest also that power laws in citation distributions, when present, account only for a very small fraction of the published papers (less than 1 % for most of science fields) and that the power-law scaling parameter (exponent) is substantially higher (from around 3.2 to around 4.7) than found in the older literature.

## Introduction

It is often argued in scientometrics, social physics and other sciences that distributions of some scientific “items” (e.g., articles, citations) produced by some scientific sources (e.g., authors, journals) have heavy tails that can be modelled using a power-law model. These distributions are then said to conform to the Lotka’s law (Lotka 1926). Examples of such distributions include author productivity, occurrence of words, citations received by papers, nodes of social networks, number of authors per paper, scattering of scientific literature in journals, and many others (Egghe 2005). In fact, power-law models are widely used in many sciences as physics, biology, earth and planetary sciences, economics, finance, computer science, and others (Newman 2005; Clauset et al. 2009). Models equivalent to Lotka’s law are known as Pareto’s law in economics (Gabaix 2009) and as Zipf’s law in linguistics (Baayen 2001). Appropriate measuring and providing scientific explanations for power laws plays an important role in understanding the behaviour of various natural and social phenomena.

This paper is concerned with empirical detection of power-law behaviour in the distribution of citations received by scientific papers. The power-law distribution of citations for the highly cited papers was first suggested by SollaPrice (1965), who also proposed a “cumulative advantage” mechanism that could generate the power-law distribution (SollaPrice 1976). More recently, a growing literature has developed that aims at measuring power laws in the right tails of citation distributions. In particular, Redner (1998), Redner (2005) found that the right tails of citation distributions for articles published in Physical Review over a century and of articles published in 1981 in journals covered by Thomson Scientific’s Web of Science (WoS) follow power laws. The latter data set was also modelled with power-law techniques by Clauset et al. (2009) and Peterson et al. (2010). The latter study also used data from 2007 list of the living highest h-index chemists and from Physical Review D between 1975 and 1994. VanRaan (2006) observed that the top of the distribution of around 18,000 papers published between 1991 and 1998 in the field of chemistry in Netherlands follows a power law distribution. Power-law models were also fitted to data from high energy physics (Lehmann et al. 2003), data for most cited physicists (Laherrère and Sornette 1998), data for all papers published in journals of the American Physical Society from 1983 to 2008 (Eom and Fortunato 2011), and to data for all physics papers published between 1980 and 1989 (Golosovsky and Solomon 2012).

Recently, Albarrán and Ruiz-Castillo (2011) tested for the power-law behavior using a large WoS dataset of 3.9 million articles published between 1998 and 2002 categorized in 22 WoS research fields. The same dataset was also used to search for the power laws in the right tail of citation distributions categorized in 219 WoS scientific sub-fields (Albarrán et al. 2011a, b). These studies offer the largest existing body of evidence on the power-law behaviour of citation distributions. Three major conclusions appear from them. First, the power-law behavior is not universal. The existence of power law cannot be rejected in the WoS data for 17 out of 22 and for 140 out of 219 sub-fields studied in Albarrán and Ruiz-Castillo (2011) and in Albarrán et al. (2011a, b), respectively. Secondly, in opposition to previous studies, these papers found that the scaling parameter (exponent) of the power-law distribution is above 3.5 in most of the cases, while the older literature suggested that the parameter value is between 2 and 3 (Albarrán et al. 2011). Third, power laws in citation distributions are rather small—on average they cover just about 2 % of the most highly cited articles in a given WoS field of science and account for about 13.5 % of all citations in the field.

The main aim of this paper is to use a statistically rigorous approach to answer the empirical question of whether the power-law model describes best the observed distribution of highly cited papers. We use the statistical toolbox for detecting power-law behaviour introduced by Clauset et al. (2009). There are two major contributions of the present paper. First, we use a very large, previously unused data set on the citation distributions of the most highly cited papers in several fields of science. This data set comes from Scopus, a bibliographic database introduced in 2004 by Elsevier, and contains 2.2 million articles published between 1998 and 2002 and categorized in 27 Scopus major subject areas of science. Most of the previous studies used rather small data sets, which were not suitable for rigorous statistical detecting of the power-law behaviour. In contrast, our sample is even bigger with respect to the most highly cited papers than the large sample used in the recent contributions based on WoS data (Albarrán and Ruiz-Castillo 2011; Albarrán et al. 2011a, b). This results from the fact that Scopus indexes about 70 % more sources compared to the WoS (López-Illescas et al. 2008; Chadegani et al. 2013) and therefore gives a more comprehensive coverage of citation distributions.^1^

The second major contribution of the paper is to provide a rigorous statistical comparison of the power-law model and a number of alternative models with respect to the problem which theoretical distribution fits better empirical data on citations. This problem of model selection has been previously studied in some contributions to the literature. It has been argued that models like stretched exponential (Laherrère and Sornette 1998), Yule (SollaPrice 1976), log-normal (Redner 2005; Stringer et al. 2008; Radicchi et al. 2008), Tsallis (Tsallis and deAlbuquerque 2000; Anastasiadis et al. 2010; Wallace et al. 2009) or shifted power law (Eom and Fortunato 2011) fit citation distributions equally well or better than the pure power-law model. However, previous papers have either focused on a single alternative distribution or used only visual methods to choose between the competing models. The present paper fills the gap by providing a systematic and statistically rigorous comparison of the power-law distribution with such alternative models as the log-normal, exponential, stretched exponential (Weibull), Tsallis, Yule and power-law with exponential cut-off. The comparison between models was performed using a likelihood ratio test (Vuong 1989; Clauset et al. 2009).

## Materials and methods

### Fitting power-law model to citation data

We follow Clauset et al. (2009) in choosing methods for fitting power laws to citation distributions. These authors carefully show that, in general, the appropriate methods depend on whether the data are continuous or discrete. In our case, the latter is true as citations are non-negative integers. Let $x$ be the number of citations received by an article in a given field of science. The probability density function (pdf) of the discrete power-law model is defined as

1 $$\begin{aligned} p(x)= \frac{x^{-\alpha }}{\zeta (\alpha ,x_0)}, \end{aligned}$$

where $\zeta (\alpha ,x_0)$ is the generalized or Hurwitz zeta function. The $\alpha$ is a shape parameter of the power-law distribution, known as the power-law exponent or scaling parameter. The power-law behaviour is usually found only for values greater than some minimum, denoted by $x_0$. In case of citation distributions, the power-law behaviour has been found on average only in the top 2 % of all articles published in a field of science (Albarrán et al. 2011a, b).

The lower bound on the power-law behaviour, $x_0$, should be therefore estimated if we want to measure precisely in which part of a citation distribution the model applies. Moreover, we need an estimate of $x_0$ if we want to obtain an unbiased estimate of the power-law exponent, $\alpha$.

We estimate $\alpha$ using the maximum likelihood (ML) estimation. The log-likelihood function corresponding to (1) is

2 $$\begin{aligned} L(\alpha ) = -n\ln \zeta (\alpha ,x_0) - \alpha \sum _{i=1}^{n}\ln x_i, \end{aligned}$$

where $x_i$ is the number of citations received by the $i\hbox {th}$ paper $(i = 1,\dots , n)$.

The ML estimate for $\alpha$ is found by numerical maximization of (2).^2^

Following Clauset et al. (2009), we use the following procedure to estimate the lower bound on the power-law behaviour, $x_{0}$. For each $x\geqslant x_{\rm min}$, we calculate the ML estimate of the power-law exponent, $\hat{\alpha }$, and then we compute the well-known Kolmogorov–Smirnov (KS) statistic for the data and the fitted model. The KS statistic is defined as

3 $$\begin{aligned} \hbox {KS} = \max \limits _{x\geqslant x_0} \left| S(x) - P(x; \hat{\alpha })\right| , \end{aligned}$$

where $S(x)$ is the cumulative distribution function (cdf) for the observations with value at least $x_0$, and $P(x,\hat{\alpha })$ is the cdf for the fitted power-law model to observations for which $x\geqslant x_0$. The estimate $\hat{x}_0$ is then chosen as a value of $x_0$ for which the KS statistic is the smallest. The standard errors for both estimated parameters, $\hat{\alpha }$ and $\hat{x}_0$, are computed with standard bootstrap methods with 1,000 replications.

### Goodness-of-fit and model selection tests

The next step in measuring power laws involves testing goodness of fit. A positive result of such a test allows to conclude that a power-law model is consistent with data. Following Clauset et al. (2009) again, we use a test based on a semi-parametric bootstrap approach.^3^ The procedure starts with fitting a power-law model to data and calculating a KS statistic (see Eq. 3) for this fit, denoted by $k$. Next, a large number of synthetic data sets is generated that follow the originally fitted power-law model above the estimated $x_{0}$ and have the same non-power-law distribution as the original data set below $\hat{x}_{0}$. Then, a power-law model is fitted to each of the generated data sets using the same methods as for the original data set, and the KS statistics are calculated. The fraction of data sets for which their own KS statistic is larger than $k$ is the *p* value of the test. It represents a probability that the KS statistics computed for data drawn from the power-law model fitted to the original data is at least as large as $k$. The power-law hypothesis is rejected if the *p* value is smaller than some chosen threshold.^4^ Following Clauset et al. (2009), we rule out the power-law model if the estimated *p* value for this test is smaller than 0.1. In the present paper, we use 1,000 generated data sets.

If the goodness-of-fit test rejects the power-law hypothesis, we may conclude that the power law has not been found. However, if a data set is fitted well by a power law, the question remains if there is an alternative distribution, which is an equally good or better fit to this data set. We need, therefore, to fit some rival distributions and evaluate which distribution gives a better fit. To this aim, we use the likelihood ratio test, which tests if the compared models are equally close to the true model against the alternative that one is closer. The test computes the logarithm of the ratio of the likelihoods of the data under two competing distributions, LR, which is negative or positive depending on which model fits data better. Specifically, let us consider two distributions with pdfs denoted by $p_1(x)$ and $p_2(x)$. The LR is defined as:

4 $$\begin{aligned} \hbox {LR} = \sum _{i=1}^{n}[\ln p_1(x_i)-\ln p_2(x_i)]. \end{aligned}$$

A positive value of the LR suggests that model $p_1(x)$ fits the data better. However, the sign of the LR can be used to determine which model should be favored only if the LR is significantly different from zero. Vuong (1989) showed that in the case of non-nested models the normalized log-likelihood ratio $\hbox {NLR}=n^{-1/2}\hbox {LR}/\sigma$, where $\sigma$ is the estimated standard deviation of LR, has a limit standard normal distribution.^5^ This result can be used to compute a *p* value for the test discriminating between the competing models. If the *p* value is small (for example, smaller than 0.1), then the sign of the LR can probably be trusted as an indicator of which model is preferred. However, if the *p* value is large, then the test is unable to choose between the compared distributions.

We have followed Clauset et al. (2009) in choosing the following alternative discrete distributions: exponential, stretched exponential (Weibull), log-normal, Yule and the power law with exponential cut-off.^6^ Most of these models have been considered in previous literature on modeling citation distribution. As another alternative, we also use the Tsallis distribution, which has been also proposed as a model for citation distributions (Wallace et al. 2009; Anastasiadis et al. 2010). Finally, we also consider a “digamma” model using exponential functions of a digamma function, which was recently introduced for distributions with heavy tails in a statistical physics framework based on the principle of maximum entropy (Peterson et al. 2013).^7^

The definitions of our alternative distributions are given in Table 1.

**Table 1:** Definitions of alternative discrete distributions

Distribution name	Probability distribution function
Exponential	$(1-\hbox {e}^{-\lambda })\hbox {e}^{\lambda x_0}\hbox {e}^{-\lambda x}$
Stretched exponential (Weibull)	$\frac{1}{\sum _{x_0}^{\infty }(q^{x^\beta } - q^{(x+1)^\beta })} q^{x^\beta } - q^{(x+1)^\beta }$
Log-normal	$\sqrt{\frac{2}{\pi \sigma ^2}}\left[ \hbox {erfc}(\frac{\hbox {ln}x_{0}-\mu }{\sqrt{2}\sigma })\right] ^{-1} \frac{1}{x}\exp \left[ -\frac{(\hbox {ln}x-\mu )^2}{2\sigma ^2} \right]$
Tsallis	$\frac{1}{\sum _{x_0}^{\infty }(1+x/\sigma )^{-\theta -1}}(1+x/\sigma )^{- \theta -1}$
Yule	$(\alpha -1)\frac{\Gamma (x_0+\alpha -1)}{\Gamma (x_0)} \frac{\Gamma (x)}{\Gamma (x+\alpha )}$
Digamma	$\frac{\hbox {e}^{-\mu k_0\psi (x+k_0)}}{\sum _{x_0}^{\infty }\hbox {e}^{-\mu k_0\psi (x+k_0)}}$
Power law with exponential cut-off	$(\sum _{x_0}^{\infty } x^{-\alpha } \hbox {e}^{-\lambda x})^{-1} x^{-\alpha }\hbox {e}^{-\lambda x}$

The distributions have been normalized to ensure that the total probability in the domain $[x_0,+\infty ]$ is 1. Discrete log-normal distribution is approximated by rounding the continuous log-normally distributed reals to the nearest integers. For Tsallis distribution, we use a parametrization considered by Shalizi (2007)

### Data

We use citation data from Scopus, a bibliographic database introduced in 2004 by Elsevier. Scopus is a major competitor to the most-widely used data source in the literature on modeling citation distributions—Web of Science (WoS) from Thomson Reuters. Scopus covers 29 million records with references going back to 1996 and 21 million pre-1996 records going back as far as 1823. An important limitation of the database is that it does not cover cited references for pre-1996 articles. Scopus contains 21,000 peer-reviewed journals from more than 5,000 international publishers. It covers about 70 % more sources compared to the WoS (López-Illescas et al. 2008), but a large part of the additional sources are low-impact journals. A recent literature review has found that the quite extensive literature that compares WoS and Scopus from the perspective of citation analysis offers mixed results (Chadegani et al. 2013). However, most of the studies suggest that, at least for the period from 1996 on, the number of citations in both databases is either roughly similar or higher in Scopus than in WoS. Therefore, is seems that Scopus constitutes a useful alternative to WoS from the perspective of modeling citation distributions.

Journals in Scopus are classified under four main subject areas: life sciences (4,200 journals), health sciences (6,500 journals), physical sciences (7,100 journals) and social sciences including arts and humanities (7,000 journals). The four main subject areas are further divided into 27 major subject areas and more than 300 minor subject areas. Journals may be classified under more than one subject area.

The analysis in this paper was performed on the level of 27 Scopus major subject areas of science.^8^ From the various document types contained in Scopus, we have selected only articles. For the purpose of comparability with the recent WoS-based studies (Albarrán and Ruiz-Castillo 2011; Albarrán et al. 2011a), only the articles published between 1998 and 2002 were considered. Following previous literature, we have chosen a common 5-year citation window for all articles published in 1998–2002.^9^ See Albarrán and Ruiz-Castillo (2011) for a justification of choosing the 5-year citation window common for all fields of science.

In order to measure the power-law behaviour of citations, we need data on the right tails of citation distributions. To this end, we have used the Scopus Citation Tracker to collect citations for $\min (100{,}000; x)$ of the highest cited articles, where $x$ is the actual number of articles published in a given field of science during 1998–2002. This analysis was performed separately for each of the 27 science fields categorized by Scopus.

Descriptive statistics for our data sets are presented in Table 2.

**Table 2:** Descriptive statistics for citation distributions, Scopus, 1998–2002, 5-year citation window

Scopus subject area of science	Total number of papers	No. of papers in the sample	% of all papers in the sample	Mean no. of citations	Std. Dev. of citations	Max. no. of citations
Agricultural and Biological Sciences	372,575	99,804	26.8	15.17	14.36	628
Arts and Humanities	47,191	47,074	99.8	1.256	3.357	91
Biochemistry, Genetics and Molecular Biology	636,421	99,819	15.7	49.09	46.29	3,118
Business, Management and Accounting	61,211	61,156	99.9	3.452	7.273	287
Chemical Engineering	158,673	98,989	62.4	7.232	9.236	344
Chemistry	416,660	99,398	23.9	21.07	21.17	1,065
Computer Science	134,179	99,933	74.5	6.44	18.13	2,737
Decision Sciences	27,409	27,393	99.9	3.467	5.496	143
Earth and Planetary Sciences	228,197	99,788	43.7	14.1	17.03	1,195
Economics, Econometrics and Finance	49,645	49,559	99.8	4.652	8.653	287
Energy	67,076	66,378	99.0	2.553	5.596	334
Engineering	439,719	99,765	22.7	11.77	15.83	971
Environmental Science	186,898	99,847	53.4	10.72	11.27	730
Immunology and Microbiology	195,339	99,858	51.1	22.11	25.11	926
Materials Science	331,310	99,591	30.1	12.48	14.49	697
Mathematics	193,740	99,922	51.6	6.912	11.38	929
Medicine	1,191,154	99,823	8.4	48.55	60.14	4,365
Neuroscience	445,181	99,886	22.4	18.97	20.39	771
Nursing	51,283	50,464	98.4	5.274	12.07	518
Pharmacology, Toxicology and Pharmaceutics	179,427	99,757	55.6	12.19	12.28	347
Physics and Astronomy	541,328	99,817	18.4	24.75	31.64	3,118
Psychology	104,449	99,736	95.5	7.446	11.55	377
Social Sciences	215,410	99,890	46.4	6.148	8.055	519
Veterinary	53,203	53,117	99.8	3.637	5.843	128
Dentistry	27,470	27,437	99.9	4.943	6.736	115
Health Professions	75,491	75,414	99.9	7.272	11.49	348
Multidisciplinary	50,287	50,226	99.9	30.38	76.08	5,187
All Sciences	6,480,926	2,203,841	34.0	14.92	27.74	5,187

In some cases, there was less than 100,000 articles published in a field of science during 1998–2002 and we were able to obtain complete or almost complete distributions of citations (see columns 2–4 of Table 2).^10^ In other cases, we have obtained only a part of the relevant distribution encompassing the right tail and some part of the middle of the distribution. The smallest portions of citation distributions were obtained for Medicine (8.4 % of total papers), Biochemistry, Genetics and Molecular Biology (15.7 %) and Physics and Astronomy (18.4 %). However, using the WoS data for 22 science categories, Albarrán and Ruiz-Castillo (2011) found that power laws account usually only for less than 2 % of the highest-cited articles. Therefore, it seems that the coverage of the right tails of citation distributions in our samples is satisfactory for our purposes.

## Results and discussion

Table 3 presents results of fitting the discrete power-law model to our data sets consisting of citations to scientific articles published over 1998–2002 (with a common 5-year citation window), separately for each of the 27 Scopus major subject areas of science. The last row gives also results for all subject areas combined (“All sciences”). Beside estimates of the power-law exponent $(\hat{\alpha })$ and the lower bound on the power-law behaviour $(\hat{x}_0)$, the table gives also the estimated number and the percentage of power-law distributed papers, as well as the *p* value for our goodness-of-fit test.

**Table 3:** Power-law fits to citation distributions, Scopus, 1998–2002, 5-year citation window

Scopus subject area of science	$\hat{x}_0$	$\hat{\alpha }$	No. of power-law papers	% of total papers	*p* value
Agricultural and Biological Sciences	92 (15.1)	4.19 (0.25)	488	0.1	0.566
Arts and Humanities	14 (5.4)	3.46 (0.47)	655	1.4	0.005
Biochemistry, Genetics and Molecular Biology	148 (28.0)	3.72 (0.13)	2,813	0.4	0.175
Business, Management and Accounting	24 (10.1)	3.4 (0.38)	1,339	2.2	0.000
Chemical Engineering	38 (6.7)	4.01 (0.19)	1,418	0.9	0.099
Chemistry	41(7.1)	3.4 (0.05)	8,193	2.0	0.110
Computer Science	26 (10.6)	2.78 (0.11)	3,989	3.0	0.000
Decision Sciences	12 (4.0)	3.36 (0.24)	1,596	5.8	0.000
Earth and Planetary Sciences	36 (8.9)	3.37 (0.09)	5,834	2.6	0.000
Economics, Econometrics and Finance	21 (10.2)	3.13 (0.36)	1,995	4.0	0.000
Energy	32 (5.4)	3.91 (0.22)	356	0.5	0.825
Engineering	26 (9.4)	3.14 (0.09)	7,986	1.8	0.000
Environmental Science	63 (10.3)	4.33 (0.22)	624	0.3	0.506
Immunology and Microbiology	78 (13.6)	3.48 (0.10)	2,713	1.4	0.049
Materials Science	43 (8.9)	3.47 (0.11)	2,687	0.8	0.193
Mathematics	24 (4.0)	3.11 (0.06)	4,152	2.1	0.012
Medicine	59 (16.3)	3.07 (0.04)	20,163	1.7	0.000
Neuroscience	135 (28.4)	4.69 (0.41)	423	0.1	0.896
Nursing	60 (15.7)	3.68 (0.40)	439	0.9	0.256
Pharmacology, Toxicology and Pharmaceutics	56 (6.8)	4.1 (0.12)	1,215	0.7	0.865
Physics and Astronomy	61 (6.5)	3.35 (0.04)	5,034	0.9	0.797
Psychology	52 (8.8)	3.9 (0.17)	1,060	1.0	0.812
Social Sciences	24 (6.4)	3.56 (0.15)	2,963	1.4	0.007
Veterinary	23 (4.0)	4.09 (0.27)	858	1.6	0.017
Dentistry	20 (2.4)	3.89 (0.18)	1,012	3.7	0.011
Health Professions	49 (10.2)	3.85 (0.24)	942	1.2	0.352
Multidisciplinary	209 (40.4)	3.24 (0.14)	1,147	2.8	0.100
All Sciences	186 (46.3)	3.45 (0.10)	6,364	0.2	0.076

Standard errors are given in parentheses

**Fig. 1:**
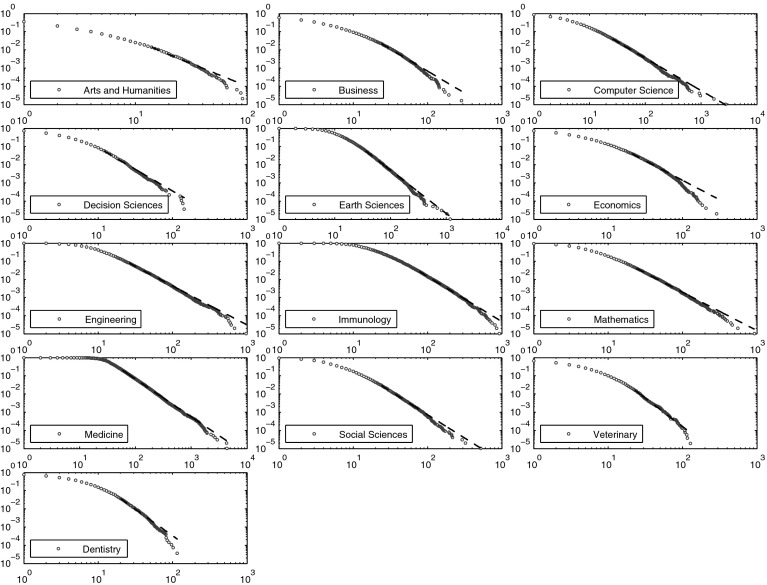
The complementary cumulative distribution functions (*blue circles*) and best power-law fits (*dashed black line*) for citation distributions that did not pass the goodness-of-fit test, Scopus, 1998–2002, 5-year citation window

Results with respect to the goodness-of-fit suggest that the power-law hypothesis cannot be rejected for the following 14 Scopus science fields: “Agricultural and Biological Sciences”, “Biochemistry, Genetics and Molecular Biology”, “Chemical Engineering”, “Chemistry”, “Energy”, “Environmental Science”, “Materials Science”, “Neuroscience”, “Nursing”, “Pharmacology, Toxicology and Pharmaceutics”, “Physics and Astronomy”, “Psychology”, “Health Professions”, and “Multidisciplinary”. The remaining 13 Scopus fields of science for which the power-law model is rejected include humanities and social sciences (“Arts and Humanities”, “Business, Management and Accounting”, “Economics, Econometrics and Finance”, “Social Sciences”), but also formal sciences (“Computer Science”, “Decision Sciences”, “Mathematics”), life sciences (“Immunology and Microbiology”, “Medicine”, “Veterinary”, “Dentistry”), as well as “Earth and Planetary Sciences” and “Engineering”. The best power-law fits for these fields of science are shown on Fig. 1.

For most of the distributions shown on Fig. 1, it can be clearly seen that their right tails decay faster than the pure power-law model indicates. This suggest that the largest observations for these distributions should be rather modeled with a distribution having a lighter tail than the pure power-law model like the log-normal or power-law with exponential cut-off models.

The *p* value for our goodness-of-fit test in case of “All Sciences” is 0.076, which is below our acceptance threshold of 0.1. However, this *p* value is non-negligible and significantly higher than *p* values for most of the 13 Scopus fields of science for which we reject the power-law hypothesis. For this reason, we conclude that the evidence is not conclusive in this case. Our result for “All Sciences” is, however, in a stark contrast with that of Albarrán and Ruiz-Castillo (2011), who using the WoS data found that the fit for a corresponding data set was very good (with a *p* value of 0.85).^11^

The estimates of the power-law exponent for the 14 Scopus science fields for which the power law seems to be a plausible hypothesis range from 3.24 to 4.69. This is in a good agreement with Albarrán and Ruiz-Castillo (2011) and confirms their assessment that the true value of this parameter is substantially higher than found in the earlier literature (Redner 1998; Lehmann et al. 2003; Tsallis and deAlbuquerque 2000), which offered estimates ranging from around 2.3 to around 3. We also confirm the observation of Albarrán and Ruiz-Castillo (2011) that power laws in citation distributions are rather small—they account usually for less than 1 % of total articles published in a field of science. The only two fields in our study with slightly “bigger” power laws are “Chemistry” (2 %) and “Multidisciplinary” (2.8 %).

The comparison between the power-law hypothesis and alternatives using the Vuong’s test is presented in Table 4. It can be observed that the exponential model can be ruled out in most of the cases. We discuss other results first for the 13 Scopus fields of science that did not pass our goodness-of-fit test. For all of these fields, except for “Veterinary”, the Yule and power-law with exponential cut-off models fit the data better than the pure power-law model in a statistically significant way. The log-normal model is better than the pure power-law model in 10 of the discussed fields; the same holds for the Weibull distribution in case of 5 fields and for the digamma distribution in case of 4 fields. However, these results do not imply that the distributions, which give a better fit to the non-power-law distributed data than the pure power-law model are plausible hypotheses for these data sets. This issue should be further studied using appropriate goodness-of-fit tests.

**Table 4:** Model selection tests for citation distributions, Scopus, 1998–2002, 5-year citation window

Scopus subject area of science	*p* value	Exponential		Weibull		Log-normal		Tsallis		Yule		Digamma		PL with cut-off	
		LR	*p*	LR	*p*	LR	*p*	LR	*p*	LR	*p*	LR	*p*	NLR	*p*
Agricultural and Biological Sciences	0.566	20.740	0.009	0.338	0.779	−0.096	0.782	0.054	0.890	−0.011	0.858	1.048	0.295	−0.268	0.464
Arts and Humanities	0.005	6.287	0.457	−6.93	0.023	−6.56	0.025	−4.325	0.189	−1.38	0.000	−1.991	0.046	−7.37	0.000
Biochemistry, Genetics and Mol. Biol.	0.175	204.5	0.000	1.22	0.758	−1.12	0.473	−1.227	0.479	−0.155	0.108	1.553	0.121	−0.567	0.287
Business, Management and Accounting	0.000	34.390	0.034	−9.60	0.013	−9.24	0.013	−7.279	0.065	−1.39	0.000	−2.072	0.038	−9.98	0.000
Chemical Engineering	0.099	69.480	0.001	−0.021	0.994	−0.972	0.480	0.025	0.990	−0.358	0.187	0.710	0.477	−0.78	0.211
Chemistry	0.110	736.0	0.000	7.48	0.262	−2.67	0.204	1.290	0.687	−0.999	0.060	3.956	0.000	−3.31	0.010
Computer Science	0.000	609.4	0.000	−7.05	0.248	−8.80	0.035	−6.719	0.132	−2.00	0.000	0.664	0.507	−5.23	0.001
Decision Sciences	0.000	77.730	0.001	−6.71	0.046	−6.81	0.048	−.0275	0.956	−2.66	0.000	−1.176	0.240	−5.91	0.001
Earth and Planetary Sciences	0.000	459.7	0.000	−4.69	0.451	−7.52	0.045	−4.928	0.264	−1.95	0.000	1.164	0.244	−5.69	0.001
Economics, Econometrics and Finance	0.000	45.080	0.021	−21.6	0.000	−20.4	0.000	−17.027	0.002	−2.68	0.000	−3.408	0.001	−22.9	0.000
Energy	0.825	20.630	0.065	0.357	0.789	−0.072	0.838	0.347	0.690	−0.023	0.884	0.813	0.416	−0.119	0.625
Engineering	0.000	825.5	0.000	–	–	−7.98	0.032	−0.763	0.877	−2.71	0.000	2.498	0.013	−7.52	0.000
Environmental Science	0.506	26.730	0.104	0.003	0.999	−0.422	0.685	−0.333	0.793	−0.114	0.334	0.303	0.762	−0.18	0.547
Immunology and Microbiology	0.049	170.3	0.000	−1.85	0.539	−2.48	0.176	−1.111	0.496	−0.268	0.076	1.643	0.100	−3.98	0.005
Materials Science	0.193	233.4	0.000	2.02	0.610	−1.02	0.460	−0.034	0.987	−0.412	0.178	1.852	0.064	−0.850	0.192
Mathematics	0.012	414.8	0.000	−1.54	0.784	−4.97	0.083	−0.264	0.943	−1.56	0.007	1.694	0.090	−5.19	0.001
Medicine	0.000	2740.0	0.000	–	–	−7.78	0.043	−4.566	0.309	−2.03	0.000	6.142	0.000	−5.62	0.001
Neuroscience	0.896	11.920	0.072	−0.018	0.987	−0.178	0.726	−0.066	0.888	−0.020	0.637	0.549	0.583	−0.285	0.451
Nursing	0.256	21.520	0.012	−0.284	0.803	−0.372	0.580	−0.048	0.936	−0.045	0.565	0.716	0.474	−0.733	0.226
Pharmacology, Toxicology and Pharm.	0.865	47.520	0.000	−0.361	0.844	−0.747	0.449	−0.002	0.999	−0.148	0.337	1.016	0.309	−1.24	0.115
Physics and Astronomy	0.797	706.2	0.000	19.5	0.006	0.048	0.646	0.954	0.495	0.091	0.771	4.514	0.000	0.000	1.000
Psychology	0.812	53.220	0.000	0.186	0.920	−0.460	0.562	0.129	0.904	−0.112	0.475	1.201	0.230	−0.791	0.208
Social Sciences	0.007	173.3	0.000	−3.56	0.366	−4.27	0.114	0.0774	0.983	−1.43	0.007	0.692	0.489	−4.21	0.004
Veterinary	0.017	38.090	0.000	0.841	0.598	−0.183	0.677	1.953	0.330	−0.047	0.874	1.520	0.128	−0.542	0.298
Dentistry	0.011	11.830	0.200	−6.60	0.025	−6.26	0.028	−3.714	0.257	−1.28	0.000	−1.958	0.050	−7.14	0.000
Health Professions	0.352	38.620	0.001	−0.944	0.599	−1.10	0.352	−0.395	0.760	−0.192	0.189	0.569	0.569	−1.63	0.071
Multidisciplinary	0.100	98.560	0.001	−1.37	0.595	−1.67	0.339	−1.497	0.377	−0.067	0.069	0.549	0.583	−1.44	0.090
All Sciences	0.076	672.3	0.000	18.30	0.009	−0.125	0.797	−0.007	0.992	−0.054	0.625	4.578	0.000	−0.240	0.488

Second column gives the *p* value for the hypothesis that the data follow a power-law model. “–” means that the maximum likelihood estimator did not converge. Positive values of the log-likelihood ratio (LR) or the normalized log-likelihood ratio (NLR) indicate that the power-law model is favored over the alternative

We now turn to results for the remaining Scopus fields of science that were not rejected by our goodness-of-fit test. The power-law hypothesis seems to be the best model only for “Physics and Astronomy”. In this case, the test statistics is always non-negative implying that the power-law model fits the data as good as or better than each of the alternatives. For the remaining 13 fields of science, the log-normal, Yule and power-law with exponential cut-off models have always higher log-likelihoods suggesting that these models may fit the data better than the pure power-law distribution. However, only in a few cases the differences between models are statistically significant. For “Chemistry” and “Multidisciplinary” both the Yule and power-law with exponential cut-off models are favoured over the pure power-law model. The power-law with exponential cut-off is also favoured in case of “Health Professions”. In other cases, the *p* values for the likelihood ratio test are large, which implies that there is no conclusive evidence that would allow to distinguish between the pure power-law, log-normal, Yule and power-law with exponential cut-off distributions. Comparing the power-law distribution with the Weibull and Tsallis distributions, we observe that the sign of the test statistic is positive in roughly half of the cases, but the *p* values are always large and neither model can be ruled out. For the considered 13 fields of science, the digamma model is never better than the power law, judging by the sign of the test statistic. Our likelihood ratio tests suggest therefore that when the power law is a plausible hypothesis according to our goodness-of-fit test it is often indistinguishable from some alternative models.

Overall, our results show that the evidence in favour of the power-law behaviour of the right-tails of citation distributions is rather weak. For roughly half of the Scopus fields of science studied, the power-law hypothesis is rejected. Other distributions, especially the Yule, power-law with exponential cut-off and log-normal seem to fit the data from these fields of science better than the pure power-law model. On the other hand, when the power-law hypothesis is not rejected, it is usually empirically indistinguishable from all alternatives with the exception of the exponential distribution. The pure power-law model seems to be favoured over alternative models only for the most highly cited papers in “Physics and Astronomy”. Our results suggest that theories implying that the most highly cited scientific papers follow the Yule, power-law with exponential cut-off or log-normal distribution may have slightly more support in data than theories predicting the pure power-law behaviour.

## Conclusions

We have used a large, novel data set on citations to scientific papers published between 1998 and 2002 drawn from Scopus to test empirically for the power-law behaviour of the right-tails of citation distributions. We have found that the power-law hypothesis is rejected for around half of the Scopus fields of science. For the remaining fields of science, the power-law distribution is a plausible model, but the differences between the power law and alternative models are usually statistically insignificant. The paper also confirmed recent findings of Albarrán and Ruiz-Castillo (2011) that power laws in citation distributions, when they are a plausible, account only for a very small fraction of the published papers (less than 1 % for most of science fields) and that the power-law exponent is substantially higher than found in the older literature.

## Footnotes

1 From the perspective of measuring power laws in citation distributions, the most important part of the distribution is the right tail. It seems that the database used in this paper has a better coverage of the right tail of citation distributions. The most highly cited paper in our database has received 5,187 citations (see Table 2), while the corresponding number for the database based on WoS is 4,461 (Li and Ruiz-Castillo 2013). Our database is further described in “Materials and methods” section.

2
Clauset et al. (2009) provide also an approximate method of estimating $\alpha$ for the discrete power-law model by assuming that continuous power-law distributed reals are rounded to the nearest integers. However, it this paper we use an exact approach based on maximizing (2).

3 If our data were drawn from a given model, then we could use the KS statistic in testing goodness of fit, because the distribution of the KS statistic is known in such a case. However, when the underlying model is not known or when its parameters are estimated from the data, which is our case, the distribution of the KS statistic must be obtained by simulation.

4 In this goodness-of-fit test we are interested in verifying if the power-law model is a plausible hypothesis for our data sets. Hence, high *p* values suggest that the power law is not ruled out. This approach is to be contrasted with the usual approach, which for a given null hypothesis interprets low *p* values as evidence in favor of the alternative hypothesis. See Clauset et al. (2009) for a more detailed discussion of these interpretations of *p* values.

5 In case of nested models, $2\hbox {LR}$ has a limit a Chi squared distribution (Vuong 1989).

6 The power-law with exponential cut-off behaves like the pure power-law model for smaller values of $x$, $x\geqslant x_0$,while for larger values of $x$ it behaves like an exponential distribution. The pure power-law model is nested within the power-law with exponential cut-off, and for this reason the latter always provides a fit at least as good as the former.

7 I would like to thank an anonymous referee for suggesting the inclusion of this distribution in our comparison of alternative models.

8 See Table 2 for a list of the analyzed Scopus areas of science.

9 For example, for articles published in 1998 we have analyzed citations received during 1998–2002, while for articles published in 2002, those received during 2002–2006.

10 For all fields of science analyzed, there were some articles with missing information on citations. These articles were removed from our samples. However, this has usually affected only about 0.1 % of our samples.

11 In Albarrán and Ruiz-Castillo (2011), the power-law hypothesis is found plausible for 17 out of 22 WoS fields of science. It is rejected for “Pharmacology and Toxicology”, “Physics”, “Agricultural Sciences”, “Engineering”, and “Social Sciences, General”. These results are not directly comparable with those of the present paper as Scopus and WoS use different classification systems to categorize journals.
